# Technical feasibility of using auditory phase-targeted stimulation after pediatric severe traumatic brain injury in an intensive care setting

**DOI:** 10.1186/s12887-022-03667-7

**Published:** 2022-10-26

**Authors:** Joëlle Ninon Albrecht, Valeria Jaramillo, Reto Huber, Walter Karlen, Christian Rainer Baumann, Barbara Brotschi

**Affiliations:** 1 Child Development Center, University Children’s Hospital Zurich, University of Zurich (UZH), Zurich, Switzerland; 2 Children’s Research Center, University Children’s Hospital Zurich, University of Zurich (UZH), Zurich, Switzerland; 3 Surrey Sleep Research Centre, Faculty of Health and Medical Sciences, University of Surrey, Guildford, UK; 4 Neuromodulation Laboratory, School of Psychology, University of Surrey, Guildford, UK; 5 Care Research and Technology Centre, UK Dementia Research Institute, at Imperial College, University of Surrey, London, Guildford, UK; 6 Department of Child and Adolescent Psychiatry and Psychotherapy, University Hospital of Psychiatry, University of Zurich (UZH), Zurich, Switzerland; 7 Mobile Health Systems Lab, Department of Health Sciences and Technology, ETH Zurich, Zurich, Switzerland; 8 Institute of Biomedical Engineering, University of Ulm, Ulm, Germany; 9 Department of Neurology, University Hospital Zurich, University of Zurich, Zurich, Switzerland; 10 Department of Neonatology and Paediatric Intensive Care, University Children’s Hospital Zurich, University of Zurich (UZH), Zurich, Switzerland

**Keywords:** Pediatric, Traumatic brain injury, PICU, EEG, Feasibility, Auditory stimulation

## Abstract

**Background:**

Supplementary treatment options after pediatric severe traumatic brain injury (TBI) are needed to improve neurodevelopmental outcome. Evidence suggests enhancement of brain delta waves via auditory phase-targeted stimulation might support neuronal reorganization, however, this method has never been applied in analgosedated patients on the pediatric intensive care unit (PICU). Therefore, we conducted a feasibility study to investigate this approach: In a first recording phase, we examined feasibility of recording over time and in a second stimulation phase, we applied stimulation to address tolerability and efficacy.

**Methods:**

Pediatric patients (> 12 months of age) with severe TBI were included between May 2019 and August 2021. An electroencephalography (EEG) device capable of automatic delta wave detection and sound delivery through headphones was used to record brain activity and for stimulation (MHSL-SleepBand version 2). Stimulation tolerability was evaluated based on report of nurses, visual inspection of EEG data and clinical signals (heart rate, intracranial pressure), and whether escalation of therapy to reduce intracranial pressure was needed. Stimulation efficacy was investigated by comparing EEG power spectra of active stimulation versus muted stimulation (unpaired *t*-tests).

**Results:**

In total, 4 out of 32 TBI patients admitted to the PICU (12.5%) between 4 and 15 years of age were enrolled in the study. All patients were enrolled in the recording phase and the last one also to the stimulation phase. Recordings started within 5 days after insult and lasted for 1–4 days. Overall, 23–88 h of EEG data per patient were collected. In patient 4, stimulation was enabled for 50 min: No signs of patient stress reactions were observed. Power spectrums between active and muted stimulation were not statistically different (all *P* > .05).

**Conclusion:**

Results suggests good feasibility of continuously applying devices needed for auditory stimulation over multiple days in pediatric patients with TBI on PICU. Very preliminary evidence suggests good tolerability of auditory stimuli, but efficacy of auditory stimuli to enhance delta waves remains unclear and requires further investigation. However, only low numbers of severe TBI patients could be enrolled in the study and, thus, future studies should consider an international multicentre approach.

## Introduction

In children and adolescents, traumatic brain injury (TBI) is the leading cause of acquired disability and death [1]. Importantly, due to the developing brain of a child, brain injuries may have a more devastating impact on children than injuries of the same severity have on adults, thus highlighting the need for additional and supplementary treatment options to improve prognosis especially in this population [2, 3]. Additionally, long reconvalescence after severe TBI and need for rehabilitation programs are associated with high healthcare costs and, thus, improving treatment after TBI is of high societal relevance [4].

Many TBI patients show electroencephalographic (EEG) slowing characterized by predominant delta waves (oscillations with a frequency of around 0.5–4 Hz; 5,6). These brain patterns are considered markers for neuroplasticity [7, 8]. Some literature describes a potential link with clearance of the brain from metabolic waste products and synaptic plasticity especially important after brain injury [9–13]. Therefore, boosting delta waves might represent a promising approach. Indeed, in animal studies (rats), interventions that enhance delta waves after TBI improved neurological and behavioural outcome [14]. Likewise, in clinical patient studies, delta waves were positively linked with outcome measures in patients after brain injury or after cardiac surgery [15–17]. However, EEG slowing has also been associated with prolonged TBI patient recovery in the clinic [5]. Taken together, evidence is thus far inconclusive: Potentially, delta waves represent neurological recovery [7] and might accordingly be more pronounced in patients with more severe brain injuries (higher need for neuronal reorganization), therefore indirectly correlating with worse outcome. However, causal evidence is scarce and, thus, experimental modulation of delta waves in the acute stage after severe TBI is needed to investigate their functional relevance. Admittedly, it is a big step from preclinical evidence to application in acute pediatrics, but direct translation potentially allows to prevent unnecessary delays.

Apart from the intention to improve neurodevelopmental outcome of children after severe TBI, a non-pharmacological approach would be very welcome. The analgosedative medication used in daily clinical practice go along with side-effects and withdrawal that should be minimized. Various non-pharmacological methods to boost delta waves are available, the most promising being auditory phase-targeted stimulation [18]: Brain activity is measured using EEG to detect delta waves. Upon recognition, brief sounds are presented targeted to the up-phase of the delta wave (up-going slope in the positive half wave). Many studies replicated efficacy of this technique to boost delta waves [18–21]. However, auditory stimulation has so far only been investigated during natural sleep. Application in the acute stage after severe TBI in analgosedated, muscle relaxed, and ventilated patients in an intensive care unit has yet to be evaluated. There is no research to this issue, therefore, little knowledge exists. Maybe our feasibility study will contribute to pave a way for an approach to investigate the potential of auditory phase targeted stimulation in this vulnerable patient group.

Therefore, as an important first step in the vision of having non-pharmacological intervention options to improve neuroplasticity, we conducted a feasibility study to investigate technical feasibility and tolerability of applying a device capable of delivering auditory stimulation in TBI patients treated in the pediatric intensive care unit (PICU). After investigating feasibility of continuously recording signals such as EEG needed for auditory stimulation over time in the PICU and adaption of the stimulation algorithm, feasibility of applying tones to enhance delta waves was examined.

## Methods

### Patients and setting

Pediatric patients (age ≥ 12 months) with severe TBI requiring management in the PICU qualified for inclusion in the study (TBI classification according to Glasgow Coma Scale score < 8; 22,23). Patients were treated according to the international guidelines by Kochanek and colleagues [24]. All these patients were comatose due to the traumatic brain injury and treated with medication for sedation, analgesia, and muscle relaxation in varying dosage depending on the patient’s needs to be in a clinically stable status without increased intracranial pressure. One legal representative was required to have good German knowledge. Exclusion criteria were known deafness, neurological or syndromal pre-existing condition, bilateral basal skull fracture, serious skin problems in the face/ear area, and fulfilment of clinical criteria of brain death within 24 h after hospital admission.

The feasibility study was conducted on a level III PICU (27 beds) of the University Children’s Hospital Zurich (Switzerland) between May 2019 and August 2021. All regulations with regards to Covid-19 safety measures were fulfilled at any time.

This feasibility study was approved by the institutional research ethics committee of ETH Zurich (EK 2019-N-18). Informed consent by the parents was obtained.

### Procedure

BB (senior PICU consultant) checked eligibility of TBI patients and informed the legal representative(s) about the study. Recordings were started as soon as possible after informed consent was obtained and ended according to patients’ situation. All patients were in supine position, head up 30 degrees. After recording phase, parents and nurses/physicians were asked about perceived disadvantages or benefits for the child due to the study (yes vs. no). Due to the challenging environment, the feasibility study was subdivided into two consecutive phases:

1. **Recording feasibility**: First, feasibility of continuously recording signals such as EEG needed for auditory stimulation (see below) over multiple days in the PICU was investigated, but auditory stimulation was muted. Study visits took place about every 24 h to exchange electrodes and devices (because of depleting batteries). Collected data was then used to configure the stimulation algorithm.
2. **Stimulation feasibility**: When adaptation of the stimulation algorithm was finished, the second phase started and feasibility of applying tones targeted to the up-phase of delta waves was examined (tolerability and efficacy of tones on delta waves). With a cautious approach, auditory stimulation was enabled for 30–60 min. Criteria to enable the stimulation: Patient is in a stable condition using treatment recommendations for management of TBI by Kochanek and colleagues [24] and shows similar brain activity as collected in the recording feasibility phase to ensure adequate stimulation algorithm configuration. Therefore, the EEG was inspected in more frequent intervals than the 24 h device exchange. Feasibility was assessed as stimulation tolerability (report of nurses, visual inspection of EEG, heart rate, intracranial pressure) and effect on EEG spectrum. Intolerability based on clinical parameters was defined as augmentation of heart rate and intracranial pressure at beginning of the stimulation and regression to baseline after stimulation offset. Auditory stimulation as every kind of stimulation may produce stress for TBI patients with consecutive increasing intracranial pressure and development of a secondary brain damage. It is crucial to prevent TBI patients of a secondary brain damage to ameliorate neurodevelopmental outcome.

### Measures and devices

Patient information was taken from patients’ charts: diagnosis, medication, sedation-agitation scale (SAS; 25) score assessed once every work shift. Additionally, for patients in the stimulation tolerability phase, heart rate and intracranial pressure were extracted from a 24 h window around the stimulation phase in one minute intervals. These clinical parameters are transferred continuously to the PDMS (patient data management system).

Electroencephalography was measured using a mobile, single-channel EEG device (MHSL-SleepBand version 2, sampling frequency 250 Hz; 26) with three self-adhesive electrodes (Ambu^®^ BlueSensor N; Ballerup, Denmark). The recording electrode was placed around the Fpz location according to the standard 10–20 system [27], but placement was individually adapted according to patients’ external injuries. Reference and ground electrodes were placed behind the ears (mastoids). The standard setup of the MHSL-SleepBand includes a headband (with embedded electrodes) and additional recording of electrooculography and electromyography. However, due to the challenging situation on the PICU, only the EEG was recorded and electrode cables were fixed with tape because the headband could not be used due to head injuries or the intracranial pressure probe.

External movement of the patients caused by medical or study staff was recorded using an actigraph placed on arm wrist or ankle depending on where the cannulas were inserted (GENEActiv^®^, Activinsights, Cambridgeshire, UK). Because the PICU represents a noisy environment that might generate confounding factors on a potential stimulation effect, we recorded in addition to the EEG several ambient parameters. Sound level (only loudness (dB), no content) was recorded with a sound level meter (UT352, UNI-T, China). Ambient light levels to characterize the artificial light that prevents natural day-night-rhythm was measured with a mobile device placed at the backside of the patients’ bed (GENEActiv^®^, Activinsights, Cambridgeshire, UK). All devices except the actigraphy to capture external movement were placed in a small container which was hanged at the backside of the patients’ bed. Additionally, an information sheet for nurses and physicians was placed next to the patients’ bed with a short study description, instructions on how to remove devices, and contact information of the study team.

### Auditory stimulation

For this phase, in-ear headphones (Sennheiser, model CX 3.00, Wedemark, Germany) were padded with cotton and fixed with tape to the patients’ ear. Auditory stimulation (50 ms 1/f pink noise bursts, volume about 53 dB SPL) was delivered in alternating 16 s ON (stimulation enabled) and 16 s OFF (stimulation disabled) windows. Pink noise is the most common acoustic stimulus in auditory stimulation studies and efficacy has been previously shown [18]. Real-time detection of delta waves and phase estimation were autonomously performed by the MHSL-SleepBand version 2 (see Ferster and colleagues [26] for technical details of the algorithms). The target phase range was in the up-going slope in the positive half-wave of the oscillation (0 to 90°).

### EEG preprocessing and statistical analysis

For all analyses, EEG data was notch filtered (50 Hz). For spectral analyses, EEG data was further filtered between 0.5 and 38 Hz and Welch’s power spectral density was estimated in 0.25 Hz bins and 20 s epochs using 4 s hanning windows (no overlap). For phase analysis of applied stimulations, EEG data was filtered between 0.5 and 2 Hz and Hilbert transformation was applied. Circular statistics are presented. EEG data of patient 4 showed pronounced cardiogenic artefacts which were removed using a previously described algorithm (EEG signal filtered between 20 and 40 Hz was used instead of a separate ECG signal; 28).

To assess stimulation effect on EEG, spectrums in ON vs. OFF windows were compared. Spectral analysis was performed in 16 s epochs of stimulation windows (no overlap 4s hanning windows) and unpaired *t*-tests were used for statistical comparison in each frequency bin.

## Results

### Patients

**Fig. 1:**
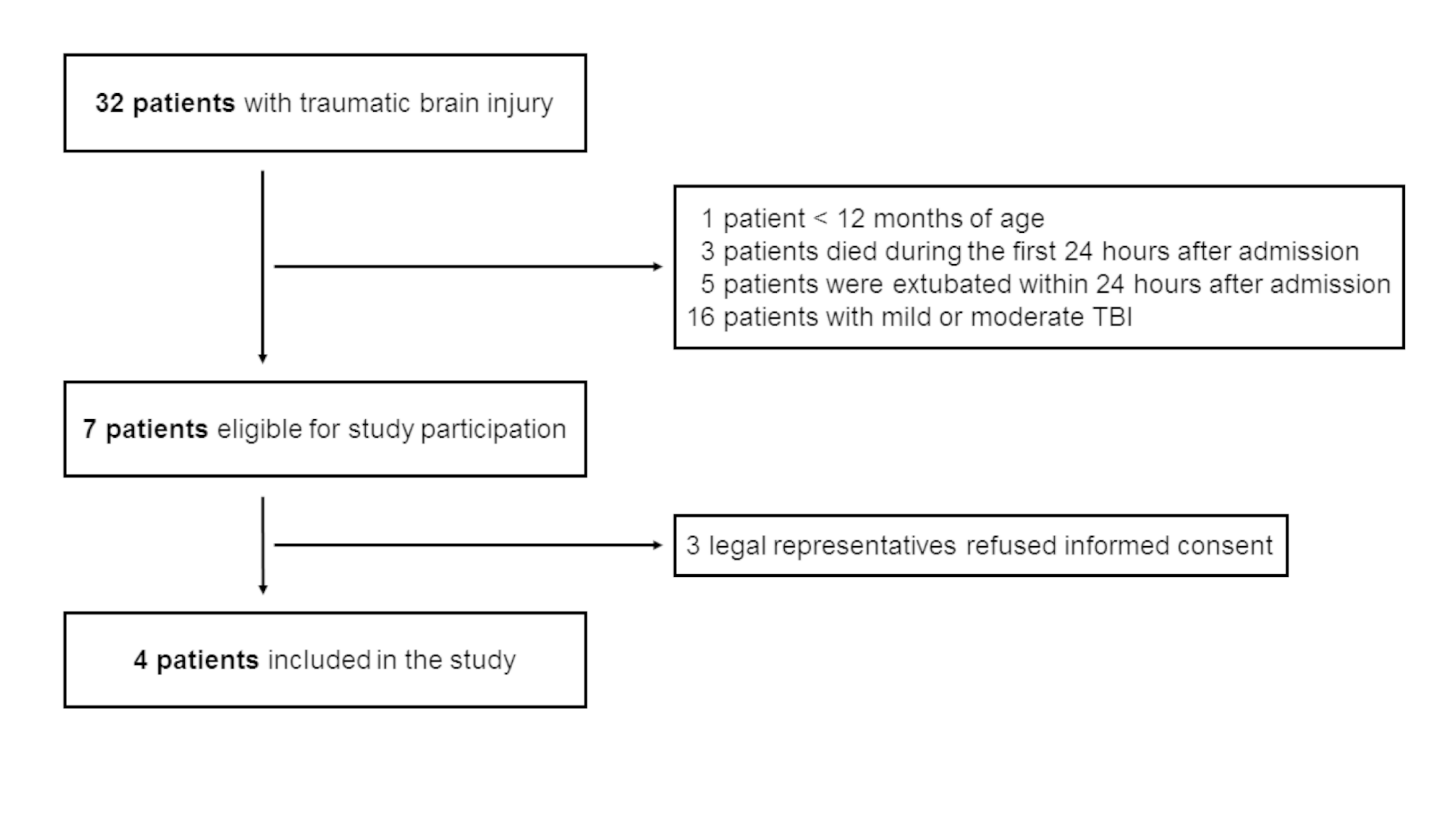
Flowchart of pediatric patients included in the study

During the study period, 3172 children were admitted to the PICU of the University Children’s Hospital Zurich. TBI was the diagnosis in 32/3172 (1%) children admitted to the PICU, 4 of them (12.5%) were included in the feasibility study (Fig. 1). Three patients were included in the recording phase and, after the stimulation algorithm was optimized to this patient group, the last patient was enrolled in the recording as well as stimulation tolerability phase. All patients were intubated and ventilated throughout study participation and received similar medication. Patient characteristics are presented in Table 1.

**Table 1:** Patient characteristics

	Age at injury (years)	Sex	TBI cause	Cerebral diagnosis	Secondary diagnoses	Medication for analgosedation	Sedation Agitation Scale (SAS)^1^	Start of recording after TBI	Length of recorded EEG data
*Patient 1*	4.3	male	fall	severe traumatic brain injury pneumocephalus bone fracture occipital	pneumonia	Dormicum Morphin Fentanyl Clonidin Rucoronium Propofol	very sedated (2) – agitated (5)	2 days	23.1 h
*Patient 2*	14	male	fall	severe traumatic brain injury shearing injuries	thoracical contusion bilateral lung contusion soft tissue emphysema laceration of the spleen, kidneys multiple fractures of different vertebras, instable pelvic fracture	Dormicum Fentanyl Rocuronium Propofol	unarousable (1) – sedated (3)	7.5 h	88 h
*Patient 3*	15.4	male	traffic accident	severe traumatic brain injury intraparenchymatoes bleeding shearing injuries orbit subarachnoidal hemorrhage multiple bone fractures frontotemporal	pneumonia	Dormicum Fentanyl Rocuronium Propofol Ketalar	very sedated (2)	4.5 days	66.4 h
*Patient 4*	14.5	female	ski accident	severe traumatic brain injury shearing injuries subdural hemorrhage		Dormicum Fentanyl Rocuronium Propofol Chloralhydrat	unarousable (1) – sedated (3)	2.5 days	77.7 h
*Notes* EEG = electroencephalography, TBI = traumatic brain injury ^1^ SAS score range during recording period (25).									

### Feasibility of applying the devices for continuous recordings over time

**Fig. 2:**
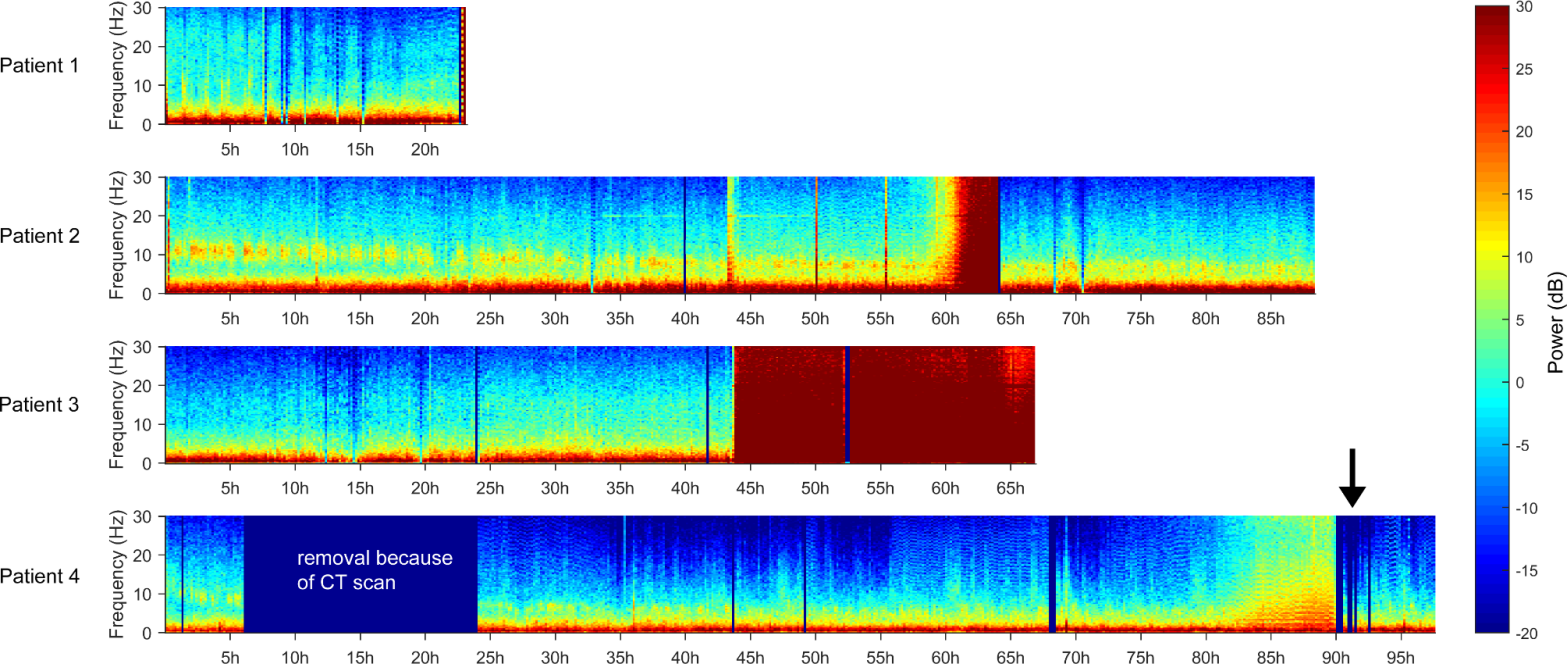
EEG spectrograms of each patient (dB from − 20 to 30, 0 dB = 1 µV^2^/Hz). Dark blue rectangular over whole frequency spectrum represent recording breaks due to device disconnection for exchange of devices and/or electrodes or necessary removal (in patient 4 for a CT scan). The arrow indicates stimulation phase in patient 4

Recordings started within 5 days after TBI impact (7.5 h – 4.5 days) and lasted between 1 and 4 days (23–88 h EEG data, Table 1). Figure 2 provides an overview over all recordings per patient. In patient 4, nurses had to remove the devices for a computer tomography scan. The study team only realized this in the following morning visit when it came to exchange the electrodes and, thus, there was a recording break of around 18 h. Otherwise, recordings were continuous with only short breaks to exchange the electrodes and devices. At certain points in time, electrode change was not possible (e.g. because patient 3 with craniectomy showed stress signals) and then, data quality deteriorated as assessed by visual inspection (in patient 2 after about 60 h, in patient 3 after about 44 h). Only in one instance (patient 4 after about 82 h), a recording (21.5 h) turned bad even though electrodes were changed. Otherwise, EEG data quality was good.

The actigraph was removed or repeatedly changed to a different location by nurses in all but one patient (patient 1). Sound level data was partly missing for two patients (1 wrong system setup (patient 1), 1 unintentional turn off (patient 2)). EEG recordings had to be terminated because of hyperactive delirium (patient 1) or bad condition (patients 2 and 3). In patient 4, the recording was ended after successful stimulation feasibility test. No device-related adverse events were reported.

### Feasibility of applying tones targeted to the up-phase of delta waves

**Fig. 3:**
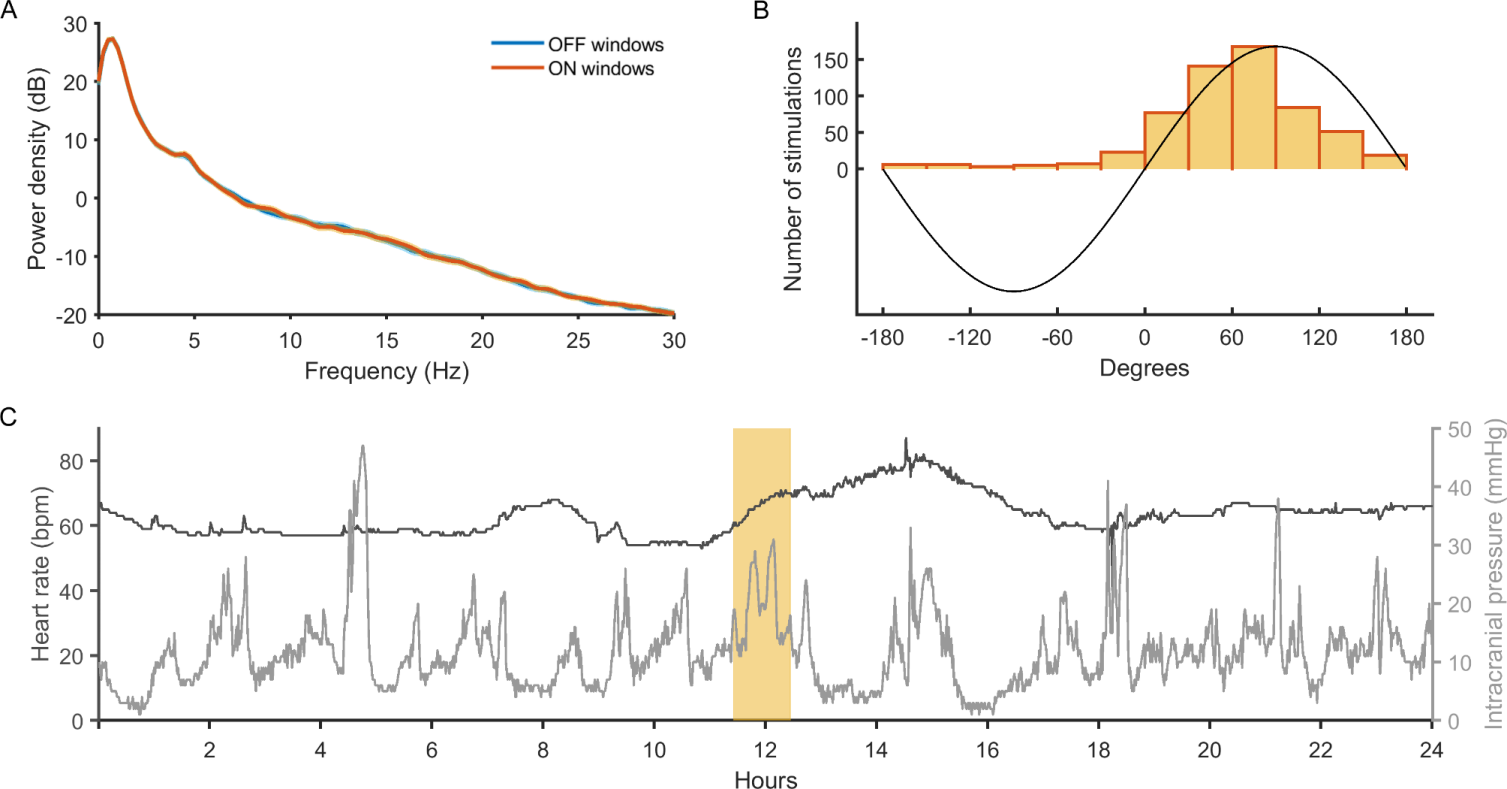
Results of the auditory stimulation test in patient 4. In a total of 92 ON windows, 590 stimulations were delivered. (A) Average +/- standard error of the mean of power density (dB from − 20 to 30, 0 dB = 1 µV^2^/Hz) in ON-windows and in OFF-windows. Power density did not differ between ON- and OFF-windows in any of the frequency bins (unpaired *t*-tests, all *P* > .05). (B) Phase distribution of all stimulations in ON-windows. Circular mean was at 66.2 degrees (standard error of the mean = 1.8). (C) Heart rate (dark grey) and intracranial pressure (light grey) before, during (rectangle), and after the stimulation phase

After 3.5 days of continuous recording in patient 4, conditions for the stimulation tolerability test were met and stimulation was applied for 51.2 min (patient deeply sedated, GCS = 3; starting at 10:30 CET). A total of 590 stimulations were administered at a mean circular phase of 66.2 (standard error of the mean = 1.8; Fig. 3, B). No signs of stress reaction were observed by the nurses. Heart rate was already increasing before stimulation started and was further increasing after stimulation had been stopped. The clinicians interpreted the tachycardia as a sign of dehydration and administered a fluid bolus. The undulation of intracranial pressure did not differ during stimulation phase (Fig. 3, C). Visual inspection of the EEG signal confirmed no arousals according to rules provided by the American Academy of Sleep Medicine.[29] Spectrums of ON- and OFF-windows were not significantly different (unpaired *t*-tests for each frequency bin, all *P*’s > 0.05; Fig. 3, A). Because there was no significant stimulation effect, sound level data was not further inspected.

### Evaluation by parents, nurses and physicians

None of the parents or nurses/physicians indicated that they thought the study led to disadvantages for the child. Two parents and three nurses/physicians out of four indicated perceived benefits for the patients.

## Discussion

Boosting delta waves might support processes such as brain plasticity and clearance of the brain from toxic waste products that have been linked with those brain patterns [7–13]. The aim of this feasibility study was to investigate feasibility of using auditory phase-targeted stimulation to enhance delta waves in pediatric patients after severe TBI treated on the PICU. To do so, continuous recording of brain activity using an EEG device capable of automatic delta wave detection and stimulation is required. Indeed, we demonstrated good feasibility of recording EEG data over multiple days in four pediatric patients (23 to 88 h of EEG data). After having verified good algorithm performance in the first three patients, stimulation was enabled for 50 min in patient 4 and preliminary evidence for good tolerability of auditory stimulation was obtained: Neither were acute clinical stress reactions reported by nurses or visible in heart rate, blood pressure or intracranial pressure, nor were arousals in the EEG. However, no significant increase of delta power was observed when stimulation was enabled compared to when it was disabled and, thus, the stimulation effect to enhance delta waves remains unclear.

Our data shows that continuous recordings allowing for auditory stimulation over several days are feasible as long as the patient is in a stable clinical condition without increased intracranial pressure. Based on our preliminary evidence it is not possible yet to define whether stable condition of the patient should remain an application criterion for auditory stimulation for future applications. All patients remained intubated and sedated throughout recording periods. Thus, whether recordings could be continued after emergence from comatose state, if not followed by hyperactive delirium, should be evaluated in a further study. The study devices did not interfere with medical procedures except for the actigraph which had to be removed or relocated in all but one patient. Therefore, actigraphy not essential for auditory stimulation should rather be omitted. For all other devices, removal was only necessary in one of the four patients who required a CT scan.

While feasibility of applying devices required for auditory stimulation was thus demonstrated and our very preliminary evidence suggests good stimulation tolerability, effect of stimulation remains unclear and requires further investigation. Thus far, at least three potential explanations for inefficacy in patient 4 are available: First, non-responders have been described as well in other studies where the approach was overall successful to boost delta waves [30]. Therefore, auditory stimulation might be effective in most, but not all TBI patients, in analogy to what has been shown in healthy sleepers. Second, on a PICU contrary to a lab setting, environment and other conditions are not well-controlled and, thus, we think that effects might be difficult to capture statistically within various other influences such as changes in medication doses and varying background noise. For the latter, evoked auditory potential protocols might provide more insights. Third, delta waves in a comatose and deeply sedated patient might simply be less modifiable than delta waves during natural sleep. This would be in line with a recent study which showed that pharmacologically enhanced delta power with sodium oxybate was not modifiable with auditory stimulation in rats [31]. Taken together, more research is required to evaluate efficacy of auditory stimulation to enhance delta waves in analgosedated TBI patients in an intensive care environment.

Nonetheless, TBI research in the PICU is a challenging intent: A 24/7 study team is required and close collaboration and exchange with PICU staff is crucial for continuous recordings over multiple days. Special attention should be drawn to electrodes and tape which have to be easily replaceable to avoid stress reactions of patients. Live visualisation of the EEG signal would enable real-time assessment of electrode connection, avoiding data loss due to bad signal quality, and, thus, a device allowing for online monitoring would be preferable. In the meantime, this has already been realized with the more integrated successor of the MHSL-SleepBand version 3 [32]. However, the biggest challenge, is the low patient numbers: In a period of over two years, only 4 out of 16 patients with severe TBI could be included in the study due to several reasons (Fig. 1). Hence, to gain sample sizes that allow for statistical evaluation of stimulation efficacy and to draw more firm conclusions, a multicentre study with long study duration, together with a flexible study team are required. An international multicentre collaboration might be needed. Taken together, investigating efficacy of auditory stimulation to enhance delta waves after severe TBI is very challenging, though of great potential in the vision of improving prognosis after pediatric TBI. Another complementary approach could be to shift auditory stimulation intervention to the post-acute phase after TBI during rehabilitation phase when medication is reduced and patients start showing natural sleep again rather than in the acute phase of the TBI when treatment on the PICU is required and patients are analgosedated.

## Conclusion

Good feasibility of performing unsupervised recordings needed for auditory stimulation in patients with severe TBI on the PICU was demonstrated. Additionally, preliminary evidence for good tolerability of auditory stimuli was obtained, but effect of sounds to modify delta waves remains unclear. Future studies should investigate similarities (and differences) between delta waves in the acute phase after severe TBI, potentially biased or even induced by analgosedation and during deep sleep, to gain more knowledge about modifiability and, in turn, potential of using auditory stimulation to improve recovery from severe TBI. For clinical studies to investigate efficacy of auditory stimulation on patient recovery, we would suggest to shift intervention to rehabilitation phase when patient has emerged from (induced) coma and shows physiological sleep.

## Data Availability

The datasets used are available from the corresponding author on reasonable request.

## Abbreviations

CT: Computer tomography
EEG: Electroencephalography
ECG: Electrocardiography
PDMS: Patient data management system
PICU: Pediatric intensive care unit
SAS: Sedation-agitation scale
SPL: Sound pressure level
TBI: Traumatic brain injury

## References

[1] Olsen M, Vik A, Lien E, Schirmer-Mikalsen K, Fredriksli O, Follestad T, et al. A population-based study of global outcome after moderate to severe traumatic brain injury in children and adolescents. J Neurosurg Pediatr. 2022;1–10. 10.3171/2021.11.PEDS21285 35061977

[2] Anderson V (2005). Functional plasticity or vulnerability after early brain injury?. Pediatrics.

[3] Hessen E, Nestvold K, Anderson V (2007). Neuropsychological function 23 years after mild traumatic brain injury: A comparison of outcome after paediatric and adult head injuries. Brain Inj.

[4] Mikkonen ED, Skrifvars MB, Reinikainen M, Bendel S, Laitio R, Hoppu S (2021). One-year costs of intensive care in pediatric patients with traumatic brain injury. J Neurosurg Pediatr.

[5] Nadlonek NA, Acker SN, Bensard DD, Bansal S, Partrick DA (2015). Early diffuse slowing on electroencephalogram in pediatric traumatic brain injury: Impact on management and prognosis. J Pediatr Surg.

[6] Steriade M, McCormick DA, Sejnowski TJ (1993). Thalamocortical oscillations in the sleeping and aroused brain. Sci (80-).

[7] Assenza G, Di Lazzaro V (2015). A useful electroencephalography (EEG) marker of brain plasticity: delta waves. Neural Regen Res.

[8] Assenza G, Pellegrino G, Tombini M, Di Pino G, Di Lazzaro V (2015). Wakefulness delta waves increase after cortical plasticity induction. Clin Neurophysiol.

[9] Iliff JJ, Lee H, Yu M, Feng T, Logan J, Nedergaard M (2013). Brain-wide pathway for waste clearance captured by contrast-enhanced MRI. J Clin Invest.

[10] Xie L, Kang H, Xu Q, Chen MJ, Liao Y, Thiyagarajan M (2013). Sleep drives metabolite clearance from the adult brain. Sci (80-).

[11] Sullan MJ, Asken BM, Jaffee MS, DeKosky ST, Bauer RM (2018). Glymphatic system disruption as a mediator of brain trauma and chronic traumatic encephalopathy. Neurosci Biobehav Rev.

[12] Fultz NE, Bonmassar G, Setsompop K, Stickgold RA, Rosen BR, Polimeni JR (2019). Coupled electrophysiological, hemodynamic, and cerebrospinal fluid oscillations in human sleep. Sci (80-).

[13] Tononi G, Cirelli C. Sleep and the price of plasticity: From synaptic and cellular homeostasis to memory consolidation and integration. Neuron [Internet]. 2014;81(1):12–34. Available from: 10.1016/j.neuron.2013.12.025. 10.1016/j.neuron.2013.12.025 PMC3921176 24411729

[14] Morawska MM, Buchele F, Moreira CG, Imbach LL, Noain D, Baumann CR (2016). Sleep modulation alleviates axonal damage and cognitive decline after rodent traumatic brain injury. J Neurosci.

[15] Mouthon AL, Meyer-Heim A, Huber R, Van Hedel HJA (2021). Neural correlates of memory recovery: Preliminary findings in children and adolescents with acquired brain injury. Restor Neurol Neurosci.

[16] Schmitt B, Finckh B, Christen S, Lykkesfeldt J, Schmid ER, Bauersfeld U (2005). Electroencephalographic changes after pediatric cardiac surgery with cardiopulmonary bypass: Is slow wave activity unfavorable?. Pediatr Res.

[17] Sarasso S, Santhanam P, Määtta S, Poryazova R, Ferrarelli F, Tononi G (2010). Non-fluent aphasia and neural reorganization after speech therapy: Insights from human sleep electrophysiology and functional magnetic resonance imaging. Arch Ital Biol.

[18] Bellesi M, Riedner BA, Garcia-Molina GN, Cirelli C, Tononi G (2014). Enhancement of sleep slow waves: Underlying mechanisms and practical consequences. Front Syst Neurosci.

[19] Cordi MJ (2021). Updated review of the acoustic modulation of sleep: Current perspectives and emerging concepts. Nat Sci Sleep.

[20] Wunderlin M, Züst MA, Hertenstein E, Fehér KD, Schneider CL, Klöppel S (2021). Modulating overnight memory consolidation by acoustic stimulation during slow-wave sleep: A systematic review and meta-analysis. Sleep.

[21] Lustenberger C, Ferster ML, Huwiler S, Brogli L, Werth E, Huber R (2022). Auditory deep sleep stimulation in older adults at home: A randomized crossover trial. Commun Med.

[22] Reilly PL, Simpson DA, Sprod R, Thomas L (1988). Assessing the conscious level in infants and young children: a paediatric version of the Glasgow Coma Scale. Child’s Nerv Syst.

[23] Teasdale G, Murray G, Parker L, Jennett B. Adding up the Glasgow Coma Score. In: Brihaye J, editor. Proceedings of the 6th European Congress of Neurosurgery. Vienna: Springer; 1988. p. 13–6.

[24] Kochanek PM, Tasker RC, Carney N, Totten AM, Adelson PD, Selden NR (2019). Guidelines for the management of pediatric severe traumatic brain injury, third edition: Update of the brain trauma foundation guidelines. Pediatr Crit Care Med.

[25] Riker RR, Picard JT, Fraser GL (1999). Prospective evaluation of the Sedation-Agitation Scale for adult critically ill patients. Crit Care Med.

[26] Ferster ML, Lustenberger C, Karlen W. Configurable mobile system for autonomous high-quality sleep monitoring and closed-loop acoustic stimulation. IEEE Sensors Lett. 2019;3(5).

[27] Jasper HH (1985). The ten-twenty electrode system of the international federation. Electroencephalogr Clin Neurophysiol.

[28] Skorucak J, Hertig-Godeschalk A, Schreier DR, Malafeev A, Mathis J, Achermann P (2020). Automatic detection of microsleep episodes with feature-based machine learning. Sleep.

[29] Berry RB, Brooks R, Gamaldo CE, Harding SM, Lloyd R, Quan SF (2017). The AASM manual for the scoring of sleep and associated events: Rules, terminology and technical specifications. Version 2.

[30] Papalambros NA, Santostasi G, Malkani RG, Braun R, Weintraub S, Paller KA (2017). Acoustic enhancement of sleep slow oscillations and concomitant memory improvement in older adults. Front Hum Neurosci.

[31] Moreira CG, Hofmann P, Müllner A, Baumann CR, Ginde VR, Kollarik S, et al. Down-phase auditory stimulation is not able to counteract pharmacologically or physiologically increased sleep depth in traumatic brain injury rats. J Sleep Res [Internet]. 2022. Available from: 10.1111/jsr.13615. 10.1111/jsr.13615 PMC9786351 35474362

[32] Ferster ML, Da Poian G, Menachery K, Schreiner SJ, Lustenberger C, Maric A, et al. Benchmarking real-time algorithms for in-phase auditory stimulation of low amplitude slow waves with wearable EEG devices during sleep. IEEE Trans Biomed Eng [Internet]. 2022. Available from: http://arxiv.org/abs/2203.02354. 10.1109/TBME.2022.3157468 35259094

